# Functional Assessment of the Stomatognathic System, after the Treatment of Edentulous Patients, with Different Methods of Establishing the Centric Relation

**DOI:** 10.1155/2018/1572037

**Published:** 2018-02-04

**Authors:** Aleksandra Nitecka-Buchta, Thomas Proba, Paulina Proba, Kamil Stefański, Stefan Baron

**Affiliations:** Department of Temporomandibular Disorders, Unit SMDZ in Zabrze, SUM in Katowice, 2 Traugutta Sq., 41-800 Zabrze, Poland

## Abstract

The study compares subjective experiences of patients, wearing complete dentures. Two different methods of determining a centric relation were performed: the traditional method using wax occlusal rims and the Gerber method, based on gothic arch tracings. The success rate of establishing a centric relation in both methods was evaluated (rentgenodiagnostics). The influence of the method used to obtain the centric relation on patients' stomatognathic system (condyle centralization, pain) was also evaluated. Better results were achieved in gothic arch tracing method. Before every prosthetic treatment of edentulous patients, a functional analysis of the TMJ is necessary. The lack of centric relation, in a long term adaptation patients, does not lead to TMD symptoms. This trial is registered with NCT03343015.

## 1. Introduction

In the field of intensive development of dental techniques, milling of finished blocks of cobalt-chromium or zirconia has almost entirely supplanted the manufacture of cast crowns and bridges. With removable dentures, the progress only applies to the cast metal framework. Methods of manufacturing complete dentures and methods of bite registration have not changed as far. In the case of partial removable dentures, we mostly register the adapted occlusion, while all the components of bite registration are only used in some cases of missing teeth. The determination of occlusion (often with the intention of increasing the vertical dimension) is finding everything from scratch, what in literature is the so-called “experience of the doctor” [1]. The matter is often complicated by the one-sided contraction of the masseter muscle that changes the rest position of the mandible, often making it impossible to determine the central relation [2]. In these cases, a preprosthetic treatment, with the goal of eliminating headaches, restoring balanced muscle tone, and establishing appropriate relations in the temporomandibular joints (TMJ), seems reasonable [3, 4].

In complicated occlusal situation, dentists often rely on the patients' subjective feelings. Occlusal splints are used mostly during night sleep. To properly evaluate the effect of the treatment, RTG images of the temporomandibular joints should be performed. First RTG of the TMD structures should be performed before prosthodontic treatment, with the mandible position, that patient is acustomed to. Second RTG should be performed after splint therapy, to compare condyles position in TMJ [5, 6].

Parafunctional activity with prolonged occlusal contacts increases masseter muscle hypertension. As a result of this hyperactivity a headache or neck pain appears, often misdiagnosed as migraine or tension type headache. Temporomandibular joint pain and muscle myalgia, which are a burden to the patient, are hard to submit to a repeated prosthetic treatment [7–9]. In the process of prosthetic treatment of edentulous patients, the most important task is establishing the proper intraarticular relation in TMJ. The risk of an undesirable error can occur, even with novel computer-assisted technologies [10]. In addition, bruxism should be confirmed or excluded, because improperly reproduced occlusion in complete dentures may lead to iatrogenic side effects. In patients with a high level of stress, a muscular-related TMD is observed more often, as well as in women [11].

**Figure 1:**
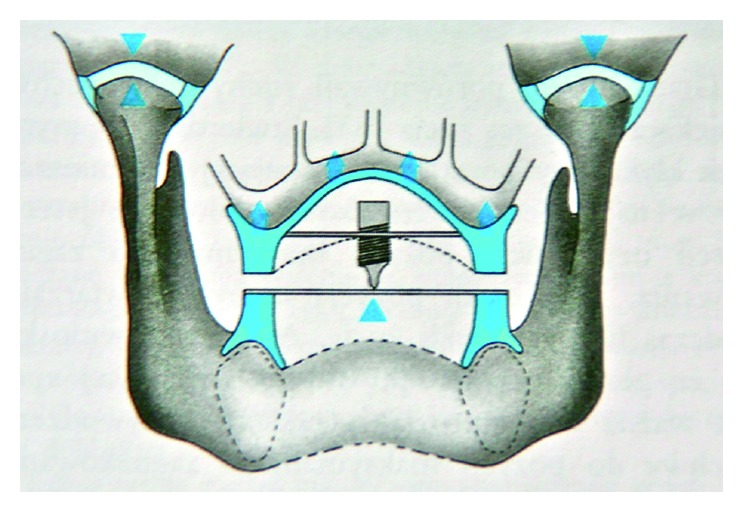
The scheme of gothic arch tracing method.

**Figure 2:**
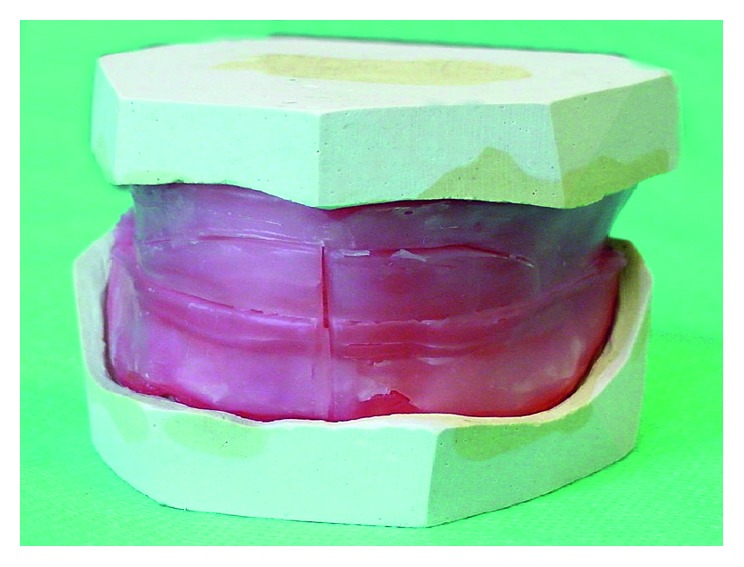
The scheme of wax occlusal rims method.

The following are the two basic definitions related to the physiological function of the masticatory system [12]:

- *Maxillo-mandibular (cranio-mandibular) relationship*: in this relationship, the condyles articulate with the thinnest avascular portion of their respective discs with the complex in the anteriosuperior position against the slopes of the articular eminences [13]. This position is independent of tooth contact. This position is clinically discernible when the mandible is directed superiorly and posteriorly. It is restricted to a purely rotary movement, about the transverse horizontal axis [14–16].
- *Maximum intercuspidation*: the cusps of the teeth of both arches fully interpose themselves with the cusps of the teeth of the opposing arch, sometimes referred to as the best fit of the teeth in position 0 mm to about 1 mm anterior from the apex of the tracing, the so-called tracing arrow point. To determine the centric relation, you can use the method of gothic arch tracings (Figure 1). Determining the centric relation with wax occlusal rims is very controversial (Figure 2). However, the occlusal vertical dimension is questionable in both methods [17].

### 1.1. Aim of the Study

The aim of the study was to determine, in retrospective, which of the methods of bite registration gives a better guarantee to guide the mandibular condyle into a centric position, in the mandibular fossa. The study also evaluates the influence of each method on the function of the stomatognathic system.

## 2. Material and Methods

Edentulous patients were enrolled to the study in The Department of TMD Zabrze, Poland and in The Zahnarztpraxis in Germany, retrospectively, after the prosthodontic therapy. They were treated by two specialists, one in Poland and one in Germany. For the study, 72 patients, who were divided into two groups, were selected. 36 people were treated with the gothic arch tracing method (group I: 23 women with an average age of 58.4) and 36 people were treated with the wax occlusal rims method (group II, 18 women with an average age of 61.7). A precise distribution of age and gender is presented in Figures 1 and 2.

Inclusion criteria were

1. temporomandibular disorder (TMD) according to RDC/TMD group I and group II,
2. edentulous patient with complete dentures,
3. patients agreement for taking part in the study.

Exclusion criteria were

1. patients addicted to analgesics drug,
2. neurological diseases with headache (migraine and cluster headache),
3. trauma in the head and neck region in past 2 years.

During the control visit (1–1.5 years after establishing a centric relation), a detailed functional assessment of stomatognathic system was performed. In anamnesis, a very special attention was given to any symptoms of headaches in the head and neck region in the past. Examination of masticatory muscles was performed: masseter muscles, neck muscle, and muscle of the upper limb. Patients with pain and headaches in the past, before the prosthodontic therapy, were supposed to mark the intensity of this pain on Visual Analogue Scale (VAS: 0–10). Severe pain was marked as 10 points in VAS scale, and no pain was marked as 0 points. Anamnestic index (AI) and Dysfunction Index (DI) were measured, according to special forms. Anamnestic index (AI) according to Helkimo was marked as

- A0 (no dysfunctional symptoms),
- A1 (medium dysfunctional symptoms),
- A2 (severe dysfunctional symptoms).

Helkimo index in a modified version was used (Di), and the clinical assessment was performed as follows:

1. Opening range: the distance between upper and lower central incisors during the opening movement was measured with the ruler:
    0 points > 40 mm
    1 point 30–39 mm
    5 points < 30 mm
2. Mandibular deviation during opening: deviation during opening movement was measured between maxillary and mandibular midline:
    0 points < 2 mm
    1 point 2–5 mm
    5 points > 5 mm
3. TMJ dysfunction observed as clicking, locking, and luxation:
    0 points < 2 mm
    1 point 2–5 mm
    5 points > 5 mm
4. TMJ pain during palpation:
    0—no pain
    1—palpable pain
    5—palpebral reflex
5. Muscle pain during bilateral palpation:
    0—no pain
    1—palpable pain
    5—palpebral reflex

The total score of each patient ranged from 0 to 25 points, and patients were classified as follows:

  0 points Di 0 = no dysfunction
  1–4 points Di I = mild dysfunction
  5–9 points Di II = moderate dysfunction
  9–25 points Di III = severe dysfunction

Collected data were analyzed with Microsoft Excel. A statistical analysis was performed. Among analyzed data, only age parameter was expressed in quotient scale. Normal distribution was verified with Shapiro-Wilk test. Results were presented in Table 1. The rest of the parameters were expressed in ordinal scale (AI, DI, and VAS) or nominal scale (gender, condyles position in TMJ, and different pain types), and nonparametrical tests such as Mann-Whitney and Chi-squared tests with Yates correction were used.

Designated *p* values were noted in figures and tables. Calculation was performed in Excel files. Student *t*-test was used for dependent variables (*p* < 0.05).

## 3. Results

In Table 1 basic age parameters of subjects in both groups were collected.

**Figure 3:**
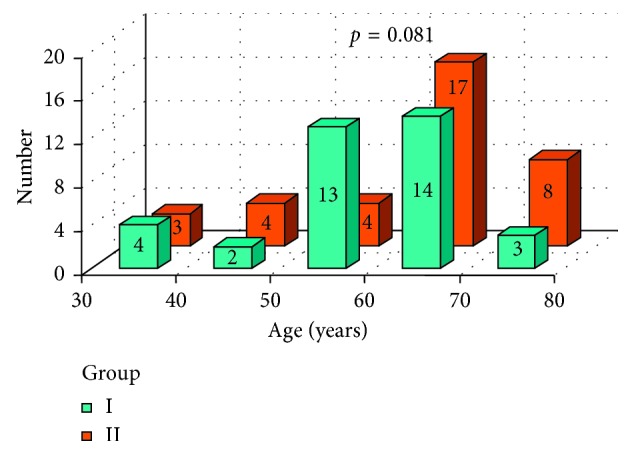
Age and result of Mann-Whitney test: comparison of both groups I and II.

**Figure 4:**
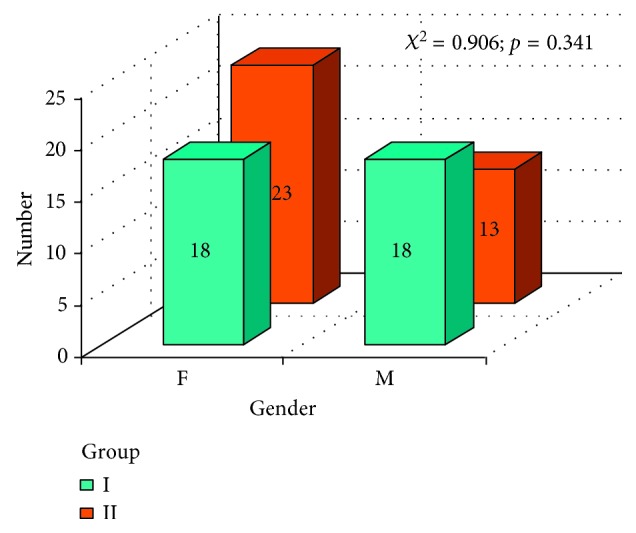
Gender and *χ* ^2^ test results, comparison of both groups I and II.

In Figure 3, age in both groups was presented and Mann-Whitney test resulted in no statistically important difference between group I and group II. The gender comparison of both groups I and II was tested with χ^2^ test. There was no statistically important difference between group I and group II (Figure 4)

**Figure 5:**
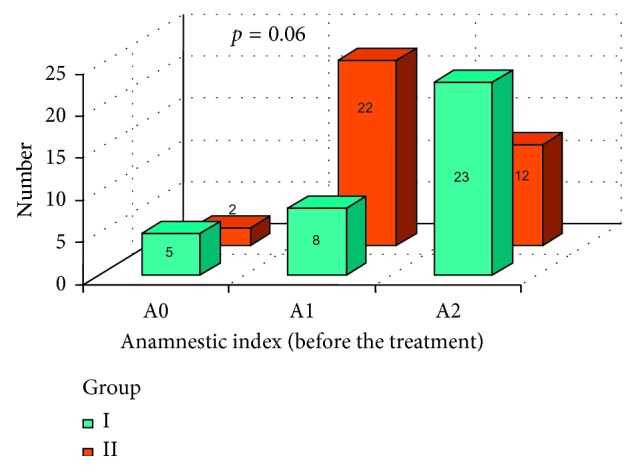
Anamnestic index (AI) in both groups before the prosthodontic treatment.

**Figure 6:**
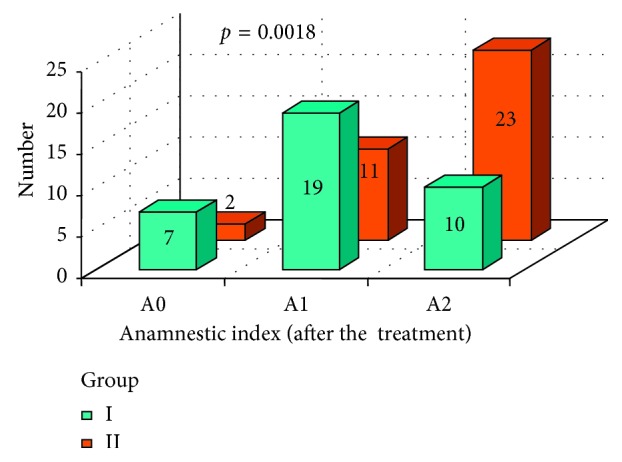
Anamnestic index (AI) in both groups I and II after the prosthodontic treatment.

In Figures 5 and 6, a subjective patients' assessment of dysfunction symptoms anamnestic index (AI) was presented, in both groups, before and after the prosthodontic treatment. Mann-Whitney test resulted in no statistically significant difference between group I and group II before the treatment Figure 5. After the therapy, a statistically important difference was observed (Figure 6) with *p*=0.0018. According to Figure 6, better results were achieved in group I.

**Figure 7:**
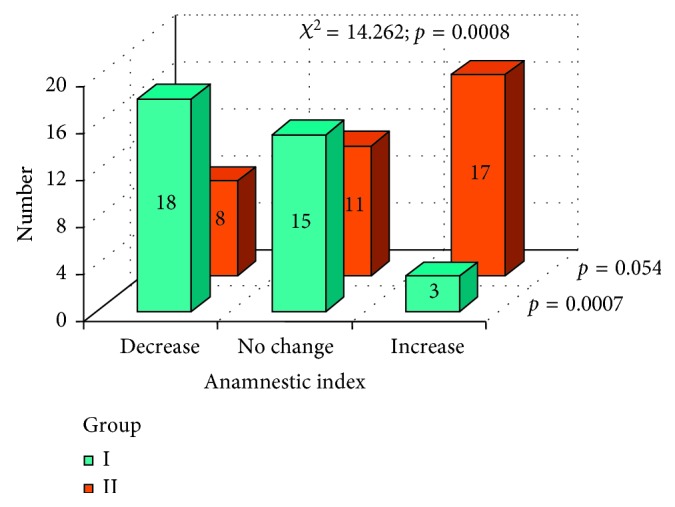
Changes in AI index after the prosthodontic therapy in both groups.

**Figure 8 fig8:**
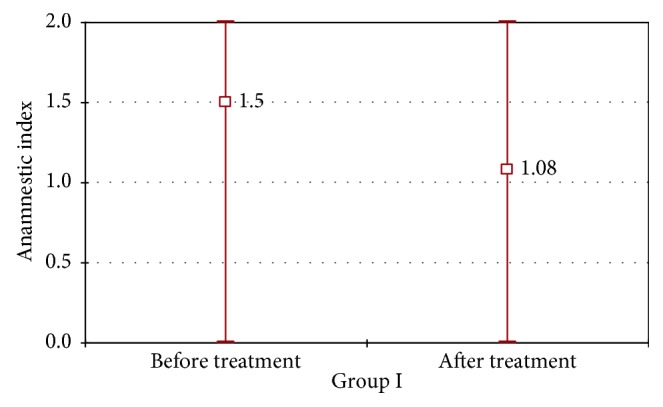
Average, minimal, and maximal values of AI index in group I before and after the prosthodontic therapy.

**Figure 9:**
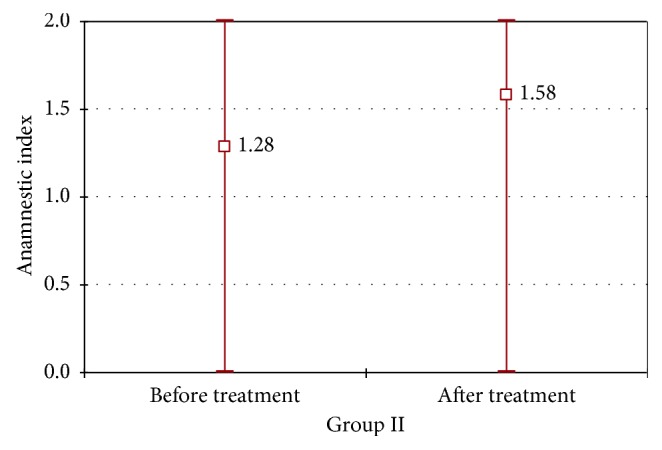
Average, minimal, and maximal values of AI index in group II before and after the prosthodontic therapy.

In Figures 7 [Fig fig8]–9, changes in AI parameter were present in both groups I and II. In group I, favorable changes of AI index were noted, with *p*=0.0007. In group II, changes in AI index were not statistically significant (*p*=0.054); however, we can observe an unfavorable tendency. A statistically significant difference between changes in AI values between group I and group II was marked with *χ* ^2^ test.

**Figure 10:**
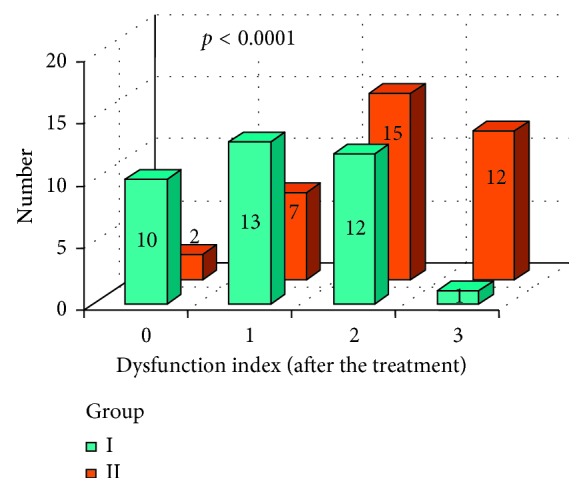
DI index values after the prosthetic therapy in group I and group II.

**Figure 11:**
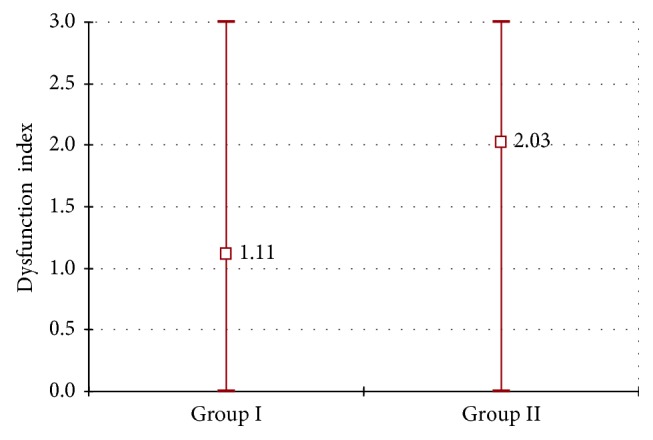
DI index average values and minimal and maximal values after the prosthetic therapy in group I and group II.

In Figures 10 and 11, dysfunction index (DI), established after the prosthodontic treatment, was presented in both groups I and II. A statistically significant difference between DI values between group I and group II was marked with *χ* ^2^ test (*p* < 0.0001). More favorable results were observed in group I.

**Figure 12:**
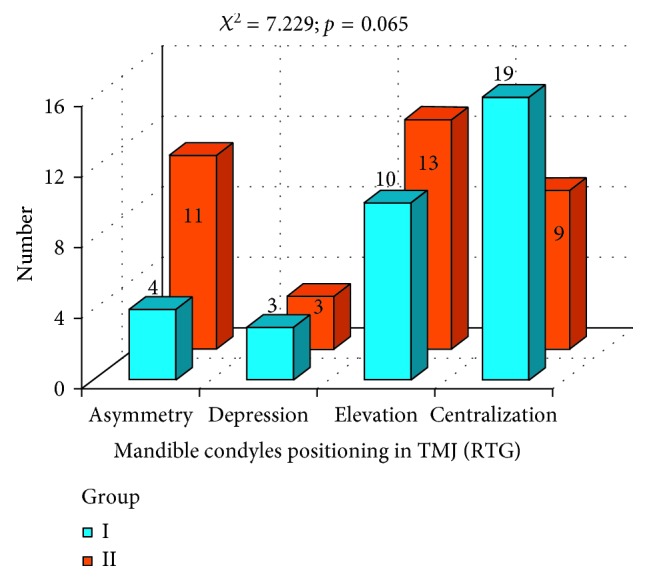
Evaluation of condyles centralization in group I and group II.

**Figure 13:**
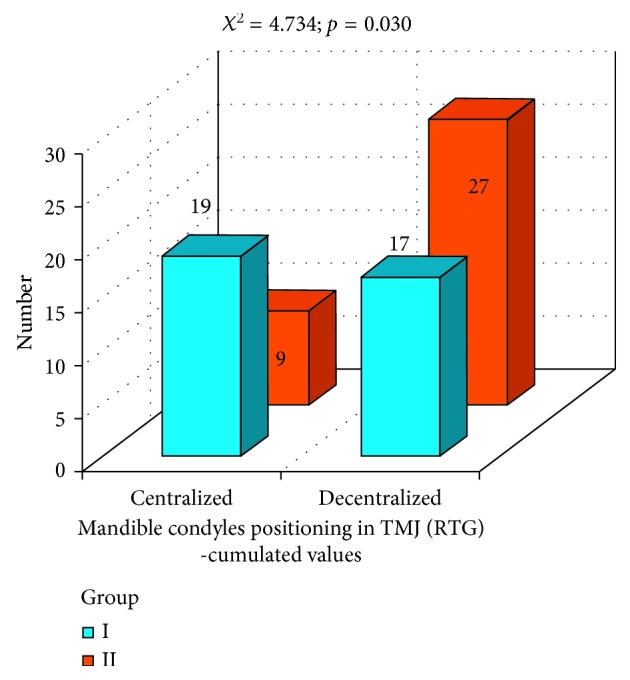
Evaluation of condyles centralization in group I and group II: condyles centralization and cumulated values for condyles decentralization.

Evaluation of condyle position in temporomandibular joints (RTG) in both groups is presented in Figure 12. Test *χ* ^2^ indicated the level of significance almost classical (*p*=0.065). The amount of centralized condyles in temporomandibular joint suggests favorable situation in group I. Additional parameter was measured: positions of condyles in the temporomandibular joint (TMJ) that were not central were cumulated together in one group (Figure 13). Obtained results suggested statistically significant difference between group I and group II (*p*=0.030). Better results in condyles centralization were achieved in group I.

**Figure 14:**
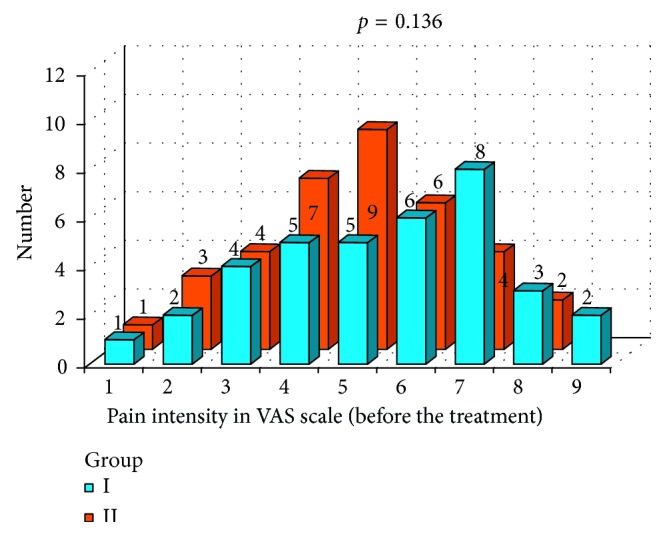
Pain intensity schedule in VAS scale before the prosthodontic treatment: group I and group II comparison.

**Figure 15:**
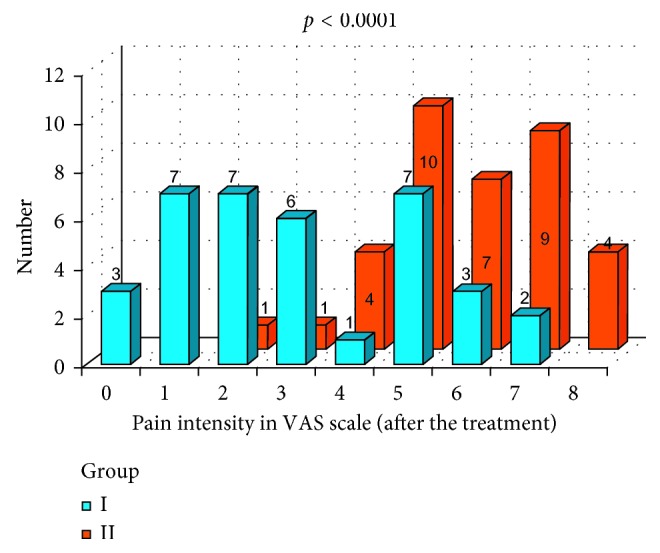
Pain intensity schedule in VAS scale after the prosthodontic treatment: group I and group II comparison.

Statistical analysis also included evaluation of pain intensity in VAS scale. In Figures 14 and 15, pain intensity schedules (in VAS scale before and after the prosthodontic therapy) for both groups are compared. Results were compared with Mann-Whitney test. Before the prosthodontic treatment, there were no statistically significant differences in pain intensity between group I and group II (*p*=0.136) (Figure 14). After the prosthodontic treatment, a statistically significant difference in pain intensity was observed between group I and group II (*p*=0.0001). Favorable analgesic effect was achieved in group I (Figure 15). Subjective pain experiences of patients in group I were recognized as lower VAS values and in group II were recognized as higher VAS values (Figure 15).

**Figure 16:**
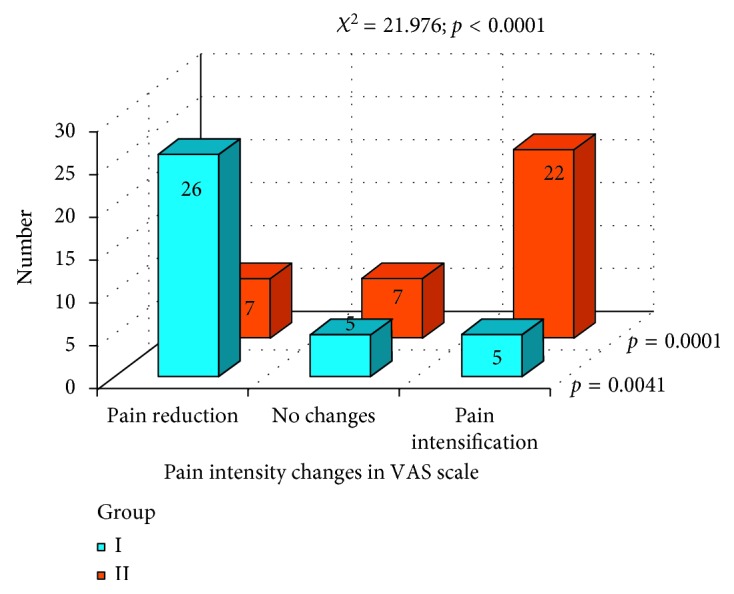
Pain intensity changes in both groups, as a result of the prosthodontic therapy.

In Figure 16 the schedules of changes in pain intensity, as a result of prosthodontic therapy, in both groups are presented. In group I (the gothic arch tracing method), a statistically significant reduction in pain intensity (VAS) was observed (*p*=0.0041). In group II (the wax rims method), a statistically significant increase in pain intensity (VAS) was marked (*p*=0.0001). The *χ* ^2^ test indicates statistically significant difference in pain intensity changes between group I and group II, after the treatment (*p* < 0.0001).

On the basis of collected data, we can conclude that changes in pain intensity in VAS scale were observed in both groups: the gothic arch tracing method (group I) and wax occlusal rims method (group II). In most cases, the prosthodontic treatment resulted in reduction of subjective pain intensity experiences (VAS scale) in patients in both the groups. The efficacy of pain reduction was better in group I—the gothic arch tracing method. During the analysis of the condyle position in TMD, the centric position was found more often in group I—the gothic arch tracing method. Establishing the centric relation with the gothic arch tracing method leads to the most posterior position of the condyles. However, it is a very problematic issue to determine the proper occlusal vertical dimension in both groups.

## 4. Discussion

**Figure 17:**
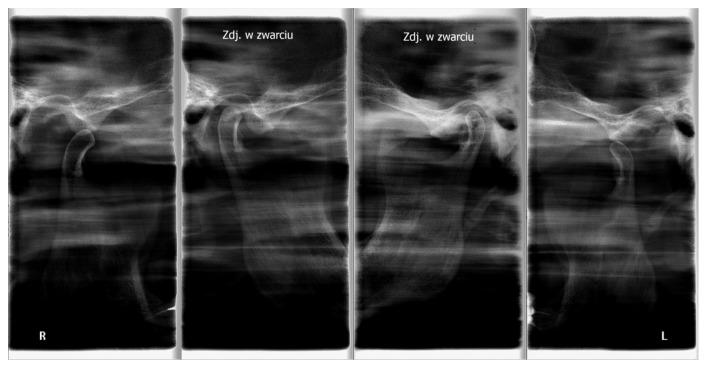
The radiographs of a nonconcentric condylar position: deficient vertical dimension (condylar position in occlusion—internal scans) and a muscle contracture in the right TMJ.

**Figure 18 fig18:**
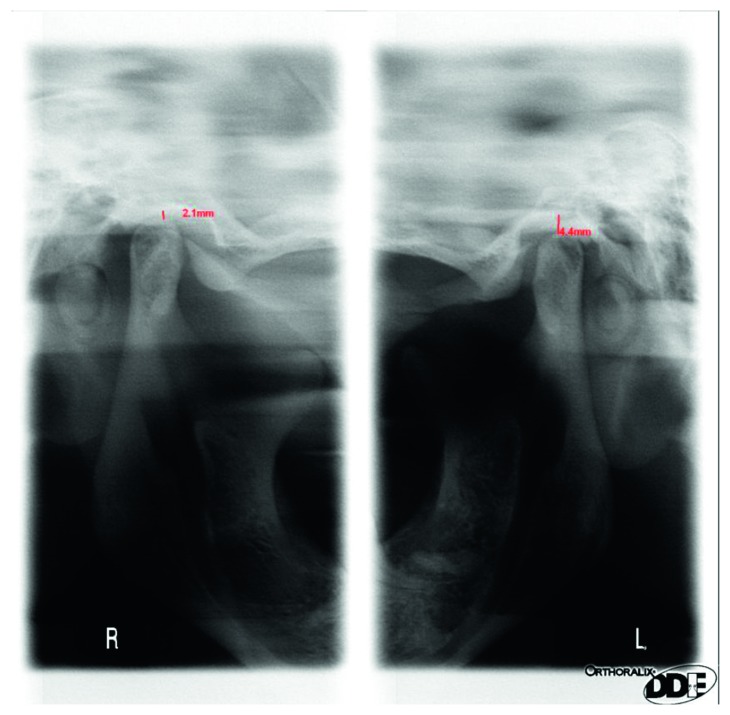
The radiographs of the TMJ and nonconcentric condylar position: in occlusion deficient vertical dimension in the right TMJ (no gap between condyle and fossa in the right TMJ, narrowed joint space).

**Figure 19:**
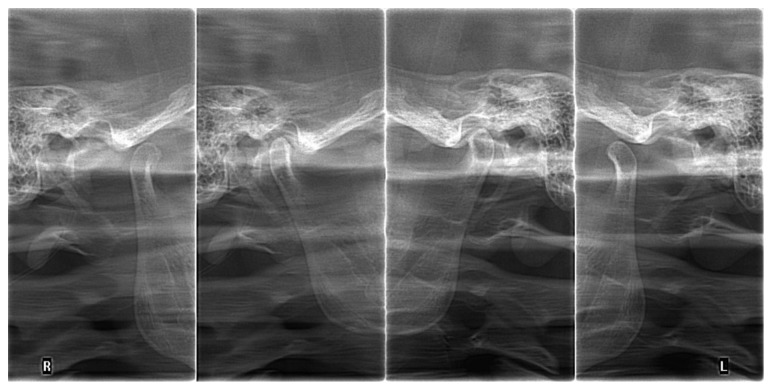
The radiographs of the TMJ with nonconcentric condylar position: an excessive left-sided occlusal vertical dimension (inferior condylar position in occlusion—internal scan, left TMJ).

Several authors have found disturbances in centric relation (CR)-maximum intercuspation (MI)-non centric position of condyles in TMJ and as an important risk factor for TMD [14, 18, 19], and others have opposed this theory [20–22]. From the achieved results, one can conclude that the physiological occlusion should be established in the centric relation because only this approach guarantees a high effectiveness in eliminating factors that disturb the normal function of the stomatognathic system [16, 23]. To establish a proper centric relation, the method of gothic arch tracings should be used routinely because it ensures a better assessment of the centric relation of the condyles in the TMJ, comparing to the method of wax occlusal rims. However, the method of the gothic arch tracings does not guarantee the correct occlusal vertical dimension. Radiographic evaluation of the TMJ (in centric relation and in maximal opening) is necessary for determining the proper occlusal vertical dimension in both methods (Figures 17 [Fig fig18]–19). Authors strongly recommend this approach. Before every prosthetic treatment of edentulous patients, a functional evaluation of the TMJ and the whole stomatognathic system is necessary. The lack of these evaluations is often followed by a symptomatic treatment of headaches in patients with TMD. The method of wax occlusal rims can even worsen the condition of the edentulous patients, with an undetected, disharmonic tension of the masseter muscles (Figures 17 and 18).

## 5. Conclusion

1. The gothic arch tracing method guarantees a better assessment of the centric relation of the condyles in the temporomandibular joints, comparing with wax occlusal rims method.
2. The wax occlusal rims method can worsen the condition of the edentulous patient with an under diagnosed, disharmonic tension of the masseter muscles.

## Figures and Tables

**Table 1:** Values of basal estimated descriptive parameters for both groups.

Group	Average values	SD standard deviation	Min	Quartile1	Median	Quartile3	Max	Test S–W
I	58.4	11.5	31.0	54.8	59.0	64.8	78.0	**<0.05**
II	61.7	11.9	35.0	58.3	64.0	68.5	78.0	>0.05
